# Supplementary figures and images for: Optimal COVID-19 testing strategy on limited resources
Source: PLoS One. 2023 Feb 24;18(2):e0281319. doi: 10.1371/journal.pone.0281319 (PMC9956024; doi:10.1371/journal.pone.0281319)

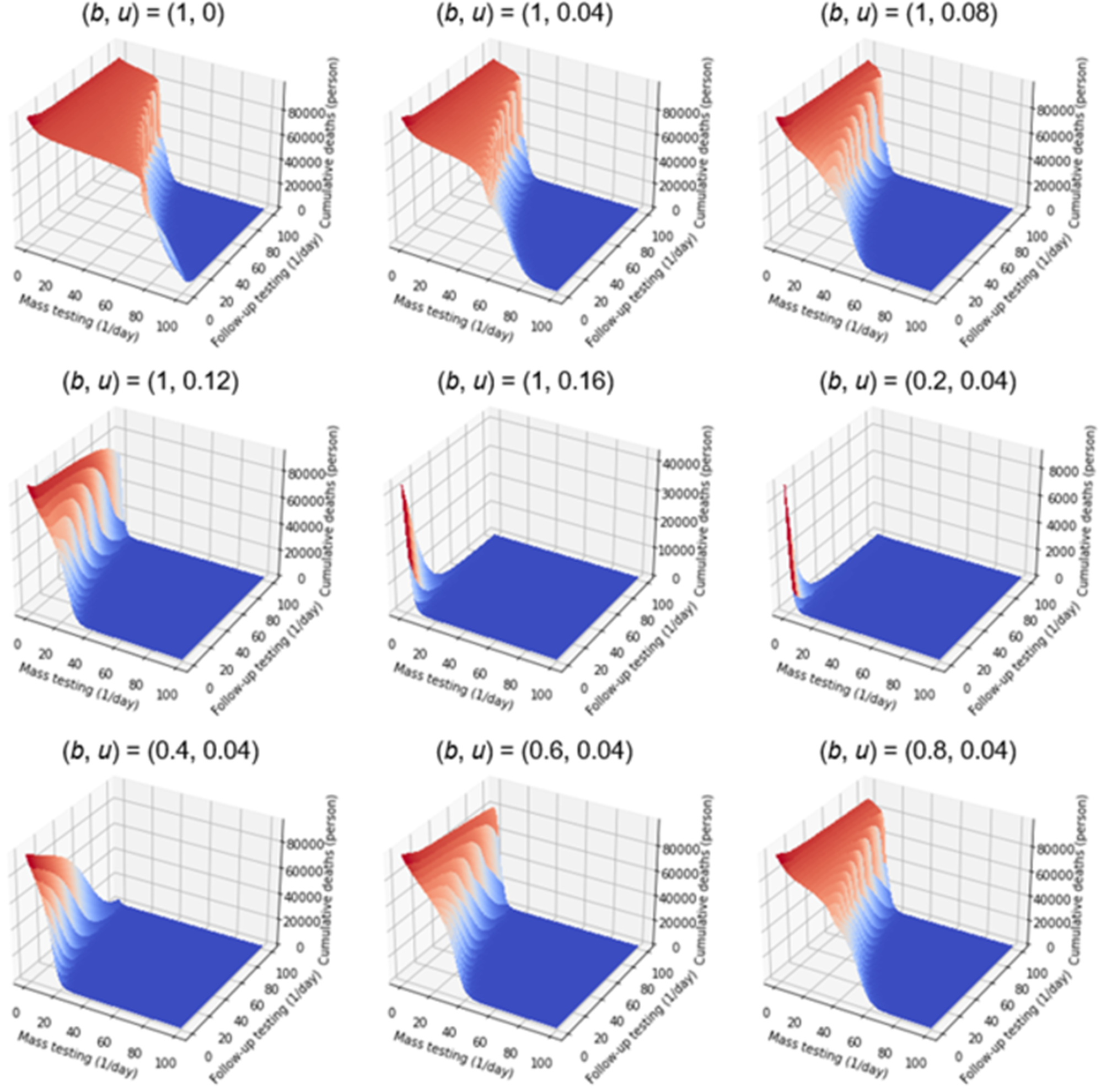

Supplement: S1 Fig — Simulations were performed using different values of b and u. (TIF) [file pone.0281319.s001.tif]

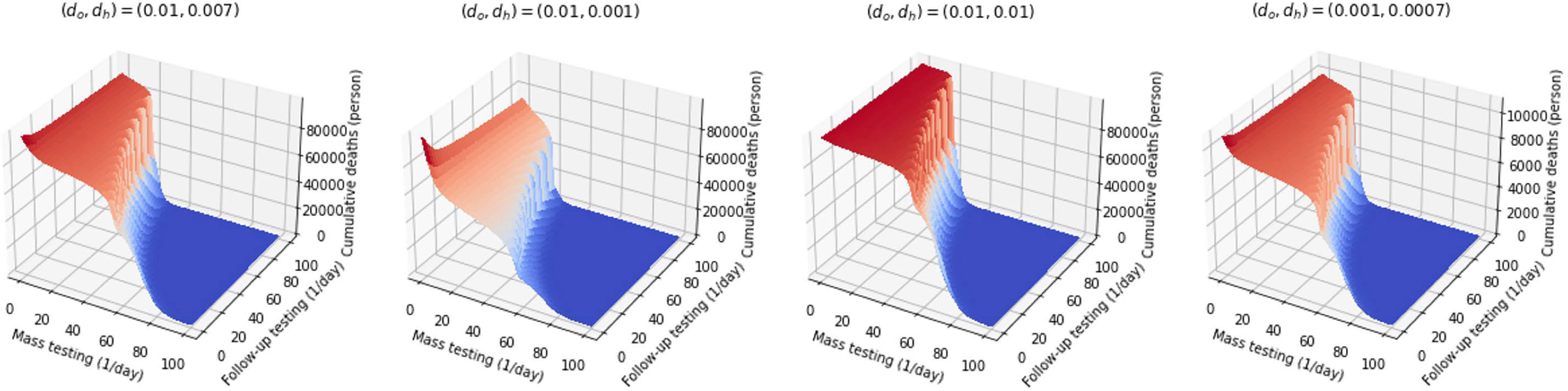

Supplement: S2 Fig — Simulations were performed using different values of do and dh, where (do, dh) of (0.01, 0.007) is a reference standard; (0.01, 0.001) means advance in treatment; (0.01, 0.01) means futile treatment; and (0.001, 0.0007) means reduction in overall mortality. (TIFF) [file pone.0281319.s002.tiff]

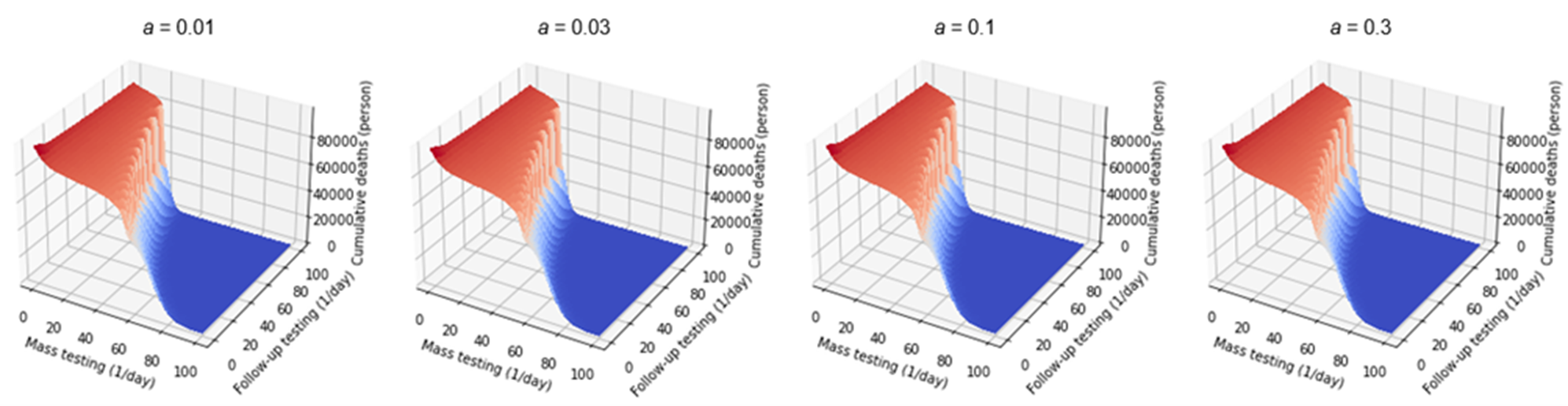

Supplement: S3 Fig — Simulations were performed using different values of parameter a. (TIF) [file pone.0281319.s003.tif]
